# Progress in the pathogenic mechanism, histological characteristics of hereditary dentine disorders and clinical management strategies

**DOI:** 10.3389/fcell.2024.1474966

**Published:** 2024-12-09

**Authors:** Qing Xue, Zhina Wu, Yinuo Zhao, Xiaoxi Wei, Min Hu

**Affiliations:** Hospital of Stomatology, Jilin University, Changchun, China

**Keywords:** hereditary dentine disorders, dentinogenesis imperfecta, dentine dysplasia, dentine sialophosphoprotein, collagen type I

## Abstract

Hereditary dentine disorders are autosomal dominant diseases that affect the development and structure of dentine, leading to various dental abnormalities and influencing the individual’s oral health. It is generally classified as dentinogenesis imperfecta (DGI) and dentine dysplasia (DD). Specifically, DGI is characterized by the abnormal formation of dentine, resulting in teeth that are discolored, translucent, and prone to fracture or wear down easily. DD is characterized by abnormal dentine development, manifested as teeth with short roots and abnormal pulp chambers, leading to frequent tooth loss. Up to now, the pathogenesis of hereditary dentine disorders has been poorly clarified and the clinical intervention is limited. Treatment for hereditary dentine disorders focuses on managing the symptoms and preventing further dental problems. Genetic counseling and testing may also be recommended as these conditions can be passed on to future generations. In this review, we summarize the clinical features, pathogenic genes, histomorphological characteristics and therapy of hereditary dentine disorders. Due to the limited understanding of the disease at present, we hope this review could improve the recognition of the disease by clinicians, stimulate more scholars to further study the deeply detailed mechanisms of the disease and explore potential therapeutic strategies, thus achieving effective, systematic management of the disease and improving the life quality of patients.

## 1 Introduction

Hereditary dentine disorders are rare genetic dental developmental disorders characterized by abnormalities in the formation and maturation of dentine, leading to structural and functional damage to the teeth (Yuan and Chen, 2023). Patients with hereditary dentine disorders typically present with teeth that are grayish-yellow or grayish-brown in appearance and are prone to wear and fractures. Dental abnormalities can cause difficulties in chewing function and even have social and psychological implications for patients (Delgado et al., 2008). The pathogenesis of this condition is associated with abnormalities in dentine synthesis and mineralization, with common causative genes including dentine sialophosphoprotein (*DSPP*), collagen type I alpha 1 (*COL1A1*), *COL1A2*, secreted modular calcium-binding protein 2 (*SMOC2*), vacuolar protein sorting 4B (*VPS4B*), and Ssu-2 homolog (*SSUH2*) (Su et al., 2023). Currently, the treatment of hereditary dentine disorders primarily focuses on restoring and protecting the teeth to alleviate symptoms and improve the quality of life via dental restorations, endodontic therapy and oral surgery. However, due to the genetic background of hereditary dentine disorders, conventional treatment methods cannot cure the disease radically. Therefore, prevention and early diagnosis is crucial for genetic counseling and family planning. Regular dental check-ups, seeking professional advice for early detection and treatment of hereditary dentine disorders, as well as implementing appropriate preventive measures, can help mitigate the impact of the disease on the patients.

In 1973, Shields classified the disease into dentinogenesis imperfecta (DGI) types I, II and III, and dentine dysplasia (DD) types I and II (Shields et al., 1973). Specifically, DGI-I is a type of osteogenesis imperfecta (OI) that affects both primary and permanent dentitions. The affected teeth show varying degrees of discoloration and wear, with genetic heterogeneity. Radiographically, they present with bulbous crowns, short and narrow roots with pulp obliteration. Affected teeth can coexist with normal teeth in the dental arch. DGI-II is commonly referred to as hereditary opalescent dentine. Compared to DGI-I, DGI-II patients exhibit complete teeth discoloration and translucency. DGI-III, also known as “Brandywine isolate” is considered a rare and severe phenotype. It is characterized by enlarged pulp chambers and root canals, with radiographic features of “shell teeth” changes. DD-I is characterized by developmental disturbances in root dentine with relatively normal crown dentine. Due to the developmental anomalies in root dentine, patients often experience delayed eruption and early exfoliation of teeth. DD-II primarily affects crown dentine development. Distinct features such as “thistle tube” pulp chambers and pulp stones are frequently observed in X-rays. The clinical features of DD-II in the primary dentition period are similar to those of DGI-II. It is reported that the incidence of DGI is between 1/6000 and 1/8000 while the incidence of DD is about 1/100,000 (Barron et al., 2008).

**FIGURE 1:**
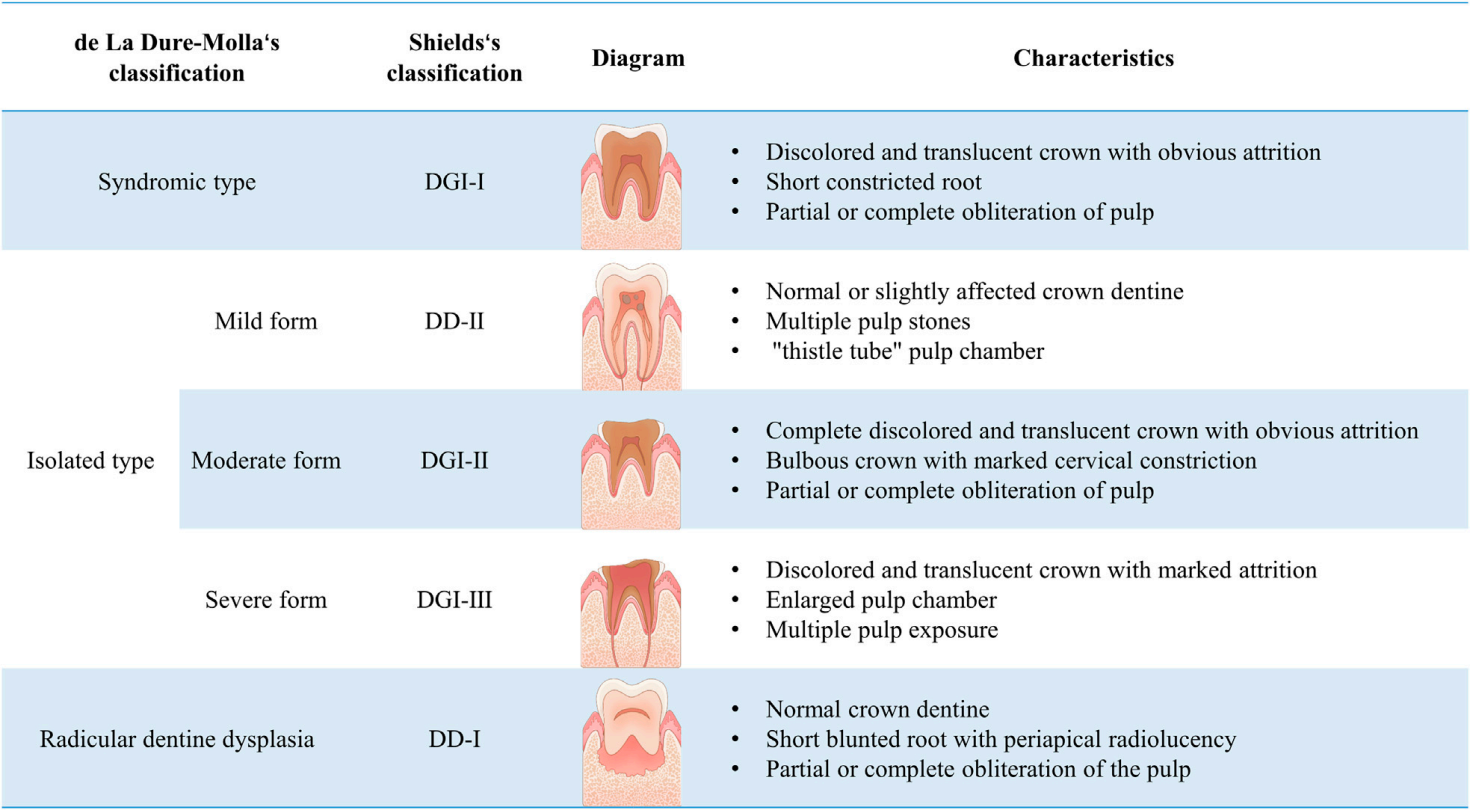
Classification and characteristics of hereditary dentine disorders.

Although Shields’ classification is widely used, it mainly relies on clinical manifestations and relatively neglects genetic factors. Therefore, based on the clinical spectrum of *DSPP* variants associated with DGI, de La Dure-Molla et al. proposed a new classification method, dividing it into isolated and syndromic types in 2015. The classification of isolated dentinogenesis disorders define DD-I as a root-type DGI, while DD-II, DGI-II, and DGI-III are defined as mild, moderate, and severe forms of DGI, respectively (shown in Figure 1) (de La Dure-Molla et al., 2015). This classification approach may help simplify the diagnosis for clinical practitioners.

## 2 Pathogenic and histological features of hereditary dentine disorders

The genetic basis of hereditary dentine disorders lies in mutations of genes encoding the main protein components of dentine. Specifically, mutations of *COL1A1* or *COL1A2* are responsible for DGI-I, while mutations in the *DSPP* are considered to be the cause of DGI-II, DGI-III and DD-II (Su et al., 2023; Yamaguti et al., 2023). It should be noted that the pathogenesis of DD-I remains unclear.

### 2.1 Dentinogenesis imperfecta type I (DGI-I)

DGI-I is a main manifestation of OI in the oral system. As a hereditary skeletal dysplasia, OI is characterized by bone fragility, skeletal deformities, as well as other connective tissue diseases such as DGI-I, hearing loss and blue sclerae with an incidence rate of 1/15000 to 1/20000 (Jovanovic et al., 2022). DGI-I is considered as a collagen-related disease due to defects of collagen type I are the main pathogenic cause (Yamaguti et al., 2023). Collagen type I, coding by *COL1A1* (17q21.31-q22,51exons) or *COL1A2* (7q22.1,52exons), is the main component of bone, dentine and other connective tissues. It is a triple-helical structure composed of two α1 chains and one α2 chain, with a Gly-X-Y tripeptide repeats (Zhang et al., 2016). Qualitative or quantitative defects in type I collagen can lead to DGI (Yamaguti et al., 2023; Andersson et al., 2017). Specifically, quantitative defects are characterized by mutations that generate premature stop codons, resulting in insufficient protein synthesis. Qualitative defects are caused by the formation of abnormal type Ⅰ procollagen fibers due to the amino acid substitutions on the α1 or α2 chains. Interestingly, mutations at different gene sites can result in varying degrees of OI ranging from mild to severe, with or without DGI (Zhang et al., 2016; Thomas and DiMeglio, 2016). While numerous mutation sites have been reported (Table 1), further research is still needed to establish the connection between specific gene mutations and the clinical manifestations associated with DGI-I.

**TABLE 1:** Mutations in the coding sequence of *COL1A1* and *COL1A2* associated with DGI-I.

Gene	Location	cDNA	Protein	Mutation class	Enamel affected	DEJ affected	References
*COL1A1*	Exon 5	c.458dupC	p.(Gly154TrpfsX15)	Frameshift	-	-	Andersson et al. (2017)
*COL1A1*	Exon 5	c.440_441insT; c.441_442insA	-	Insertion	No obvious changes	Wider	Duan et al. (2016)
*COL1A1*	Exon 6	c.484C>T	p.(Gln162X)	Nonsense	-	-	Zhang et al. (2016)
*COL1A1*	Exon 7	c.573_574delCCinsG	p.(Pro193LeufsX72)	Frameshift	-	-	Zhang et al. (2016)
*COL1A1*	Exon 7	c.579delT	p.(Gly194ValfsX71)	Frameshift	-	-	Andersson et al. (2017)
*COL1A1*	Exon 8	c.589G>T	p.(Gly197Cys)	Missense	-	-	Andersson et al. (2017)
*COL1A1*	Exon 8	c.643-2A>G	Splice-2A>G	Splice site	-	-	Zhang et al. (2016)
*COL1A1*	Exon 11	c.752G>A	p.(Gly251Asp)	Missense	-	-	Andersson et al. (2017)
*COL1A1*	Exon 11	c.757C>T	p.(Arg253X)	Nonsense	-	-	Zhang et al. (2016)
*COL1A1*	Exon 11	c.769G>A	p.(Gly257Arg)	Missense	-	-	Zhang et al. (2016)
*COL1A1*	Exon 12	c.851G>C	p.(Gly284Ala)	Missense	-	-	Andersson et al. (2017)
*COL1A1*	Exon 15	c.972_978dup	p.(Ala327X)	Nonsense	-	-	Andersson et al. (2017)
*COL1A1*	Exon 15	c.977G>A	p.(Gly326Asp)	Missense	-	-	Andersson et al. (2017), Malmgren et al. (2017)
*COL1A1*	Exon 15	c.994G>A	p.(Gly332Arg)	Missense	-	-	Andersson et al. (2017), Malmgren et al. (2017)
*COL1A1*	Exon 17	c.1057G>A	p.(Gly353Ser)	Missense	-	-	Andersson et al. (2017), Malmgren et al. (2017)
*COL1A1*	Exon 17	c.1121G>C	P.(Gly374Ala)	Missense	-	-	Zhang et al. (2016)
*COL1A1*	Exon 17	c.1127dupC	p.(Gly377TrpfsX15)	Frameshift	-	-	Andersson et al. (2017)
*COL1A1*	Exon 19	c.1201G>A	p.(Gly401Ser)	Missense	-	-	Andersson et al. (2017), Malmgren et al. (2017)
*COL1A1*	Exon 19	c.1292delG	p.(Gly431ValfsX110)	Frameshift	-	-	Andersson et al. (2017)
*COL1A1*	Exon 22	c.1463G>C	p.(Gly488Ala)	Missense	No obvious changes	-	Zeng et al. (2021)
*COL1A1*	Exon 23	c.1522G>A	p.(Ala508Thr)	Missense	-	-	Zhang et al. (2016)
*COL1A1*	Exon 23	c.1588G>A	p.(Gly530Ser)	Missense	-	-	Zhang et al. (2016)
*COL1A1*	Exon 25	c.1678G>T	p.(Gly560Cys)	Missense	-	-	Zhang et al. (2016)
*COL1A1*	Exon 25	c.1678G>A	p.(Gly560Ser)	Missense	-	-	Andersson et al. (2017), Malmgren et al. (2017)
*COL1A1*	Intron 26	c.1821 + 1G>A	Splice+1G>A	Splice site	-	-	Andersson et al. (2017)
*COL1A1*	Exon 28	c.1930-2A>C	Splice-2A>C	Splice site	-	-	Zhang et al. (2016)
*COL1A1*	Exon 31	c.2075G>C	p.(Gly692Ala)	Missense	-	-	Andersson et al. (2017), Malmgren et al. (2017)
*COL1A1*	Exon 31	c.2089C>T	p.(Arg697X)	Nonsense	-	-	Andersson et al. (2017)
*COL1A1*	Exon 31	c.2110G>T	p.(Gly704Cys)	Missense	-	-	Andersson et al. (2017), Malmgren et al. (2017)
*COL1A1*	Exon 32	c.2155G>A	p.(Gly719Ser)	Missense	-	-	Andersson et al. (2017)
*COL1A1*	Intron 32	c.2235 + 1G>A	Splice+1G>A	Splice site	-	-	Andersson et al. (2017), Malmgren et al. (2017)
*COL1A1*	Exon 33_34	c.2297C>G	p.(Thr766Ser)	Missense	-	-	Zhang et al. (2016)
*COL1A1*	Exon 33_34	c.2299G>A	p.(Gly767Ser)	Missense	-	-	Zhang et al. (2016)
*COL1A1*	Exon 33_34	c.2335G>A	p.(Gly779Ser)	Missense	-	-	Andersson et al. (2017)
*COL1A1*	Exon 36	c.2450delC	p.(Pro817LeufsX291)	Frameshift	-	-	Zhang et al. (2016)
*COL1A1*	Exon 37	c.2461G>A	p.(Gly821Ser)	Missense	-	-	Zhang et al. (2016), Andersson et al. (2017), Malmgren et al. (2017)
*COL1A1*	Exon 37	c.2515G>A	p.(Gly839Ser)	Missense	-	-	Andersson et al. (2017), Malmgren et al. (2017)
*COL1A1*	Exon 37	c.2525delG	p.(Gly842AlafsX266)	Frameshift	-	-	Andersson et al. (2017)
*COL1A1*	Exon 38	c.2596G>A	p.(Gly866Ser)	Missense	-	-	Andersson et al. (2017), Malmgren et al. (2017)
*COL1A1*	Exon 41	c.2867G>C	p.(Gly956Ala)	Missense	-	-	Zhang et al. (2016)
*COL1A1*	Exon 42	c.3026delC	p.(Pro1009LeufsX99)	Frameshift	-	-	Andersson et al. (2017)
*COL1A1*	Exon 44	c.3118G>A	p.(Gly1040Ser)	Missense	-	-	Andersson et al. (2017), Malmgren et al. (2017)
*COL1A1*	Intron 44	c.3208-6C>T	Splice-6C>T	Splice site	-	-	Andersson et al. (2017)
*COL1A1*	Exon 45	c.3226G>A	p.(Gly1076Ser)	Missense	-	-	Andersson et al. (2017), Malmgren et al. (2017)
*COL1A1*	Exon 45	c.3235G>A	p.(Gly1079Ser)	Missense	-	-	Andersson et al. (2017)
*COL1A1*	Intron 3	c.333 + 2T>C	Splice+2T>C	Splice site	-	-	Andersson et al. (2017)
*COL1A1*	Intron 47	c.3424-6C>G	Splice-5G>C	Splice site	-	-	Andersson et al. (2017), Malmgren et al. (2017)
*COL1A1*	Exon 48	c.3505G>A	p.(Gly1169Ser)	Missense	-	-	Andersson et al. (2017), Malmgren et al. (2017)
*COL1A1*	Exon 49	c.3567delT	p.(Gly1190ValfsX49)	Frameshift	-	-	Andersson et al. (2017)
*COL1A1*	Exon 49	c.3790A>G	p.(Met1264Val; Met1264GlufsX59)	Frameshift	-	-	Andersson et al. (2017)
*COL1A1*	Exon 50	c.3815G>T	p.(Gly1272Val)	Missense	-	-	Andersson et al. (2017)
*COL1A1*	Exon 52	c.4386delC	p.(Phe1463SerfsX63)	Frameshift	-	-	Andersson et al. (2017)
*COL1A2*	Exon 1	c.12T>G	p.(Phe4Leu)	Missense	-	-	Zhang et al. (2016)
*COL1A2*	Exon 16	c.752C>T	p.(Ser251Phe)	Missense	Reduced elastic modulus	Irregular	Intarak et al. (2021)
*COL1A2*	Exon 16	c.758G>T	p.(Gly253Val)	Missense	Reduced elastic modulus	Irregular	Intarak et al. (2021)
*COL1A2*	Exon 17	c.793G>C	p.(Gly265Arg)	Missense	-	-	Andersson et al. (2017)
*COL1A2*	Exon 17	c.794G>A	p.(Gly265Asp)	Missense	-	-	Andersson et al. (2017)
*COL1A2*	Exon 17	c.795G>A	p.(Gly265Arg)	Missense	-	-	Malmgren et al. (2017)
*COL1A2*	Exon 17	c.812G>A	p.(Gly271Asp)	Missense	-	-	Zhang et al. (2016)
*COL1A2*	Exon 17	c.856G>A	p.(Gly286Ser)	Missense	-	-	Andersson et al. (2017), Malmgren et al. (2017)
*COL1A2*	Exon 19	c.992G>A	p.(Gly331Asp)	Missense	-	-	Andersson et al. (2017), Malmgren et al. (2017)
*COL1A2*	Exon 19	c.1009G>A	p.(Gly337Ser)	Missense	-	-	Zhang et al. (2016), Andersson et al. (2017), Malmgren et al. (2017)
*COL1A2*	Exon 21	c.1162G>C	p.(Gly388Arg)	Missense	-	-	Andersson et al. (2017), Malmgren et al. (2017)
*COL1A2*	Exon 21	c.1171G>A	p.(Gly391Ser)	Missense	Glass-like appearance	-	Andersson et al. (2017), Malmgren et al. (2017), Lee et al. (2021), Kantaputra et al. (2018a)
*COL1A2*	Intron 21	c.1197 + 5G>A	Splice+5G>A	Splice site	-	-	Andersson et al. (2017), Malmgren et al. (2017)
*COL1A2*	Exon 23	c.1268G>A	p.(Arg423His)	Missense	-	-	Andersson et al. (2017)
*COL1A2*	Exon 25	c.1406G>C	p.(Gly469Ala)	Missense	-	-	Andersson et al. (2017), Malmgren et al. (2017)
*COL1A2*	Exon 25	c.1451G>A	p.(Gly484Glu)	Missense	-	-	Kantaputra et al. (2018b)
*COL1A2*	Exon 26	c.1531G>T	p.(Gly511Cys)	Missense	No obvious changes	-	Nutchoey et al. (2021)
*COL1A2*	Exon 31	c.1801G>A	p.(Gly601Ser)	Missense	-	-	Andersson et al. (2017), Malmgren et al. (2017)
*COL1A2*	Exon 32	c.1937G>T	p.(Gly646Val)	Missense	-	-	Andersson et al. (2017), Malmgren et al. (2017)
*COL1A2*	Exon 34	c.2027G>T	p.(Gly676Val)	Missense	No obvious changes	-	Nutchoey et al. (2021)
*COL1A2*	Exon 34	c.2081G>A	p.(Gly694Asp)	Missense	-	-	Zhang et al. (2016)
*COL1A2*	Intron 43	c.2835 + 1G>A	Splice+1G>A	Splice site	-	-	Andersson et al. (2017), Malmgren et al. (2017)
*COL1A2*	Exon 44	c.2918G>T	p.(Gly973Val)	Missense	-	-	Andersson et al. (2017)
*COL1A2*	Exon 46	c.3008G>A	p.(Gly1003Asp)	Missense	-	-	Andersson et al. (2017), Malmgren et al. (2017)
*COL1A2*	Exon 46	c.3034G>A	p.(Gly1012Ser)	Missense	-	-	Andersson et al. (2017)
*COL1A2*	Exon 46	c.3089G>C	p.(Gly1030Ala)	Missense	-	-	Andersson et al. (2017), Malmgren et al. (2017)
*COL1A2*	Exon 47	c.3106G>C	p.(Gly1036Arg)	Missense	-	-	Andersson et al. (2017), Malmgren et al. (2017)
*COL1A2*	Exon 48	c.3197G>T	p.(Gly1066Val)	Missense	-	-	Zhang et al. (2016)
*COL1A2*	Exon 48	c.3233G>A	p.(Gly1078Asp)	Missense	-	-	Lee et al. (2021)
*COL1A2*	Exon 49	c.3296G>A	p.(Gly1099Glu)	Missense	Enamel pits and grooves	Irregular	Budsamongkol et al. (2019)

(“-” refers to not mentioned in the corresponding literature).

The histological structure of the affected dentine in DGI-I showed remarkable abnormalities. Scanning electron microscopy (SEM) revealed a reduction and disarrangement of dentinal tubules, rough dentine texture, large holes, and ectopic calcification masses (Kantaputra et al., 2018b; Nutchoey et al., 2021). Histologically, the dentine of DGI-I exhibited a decreased quantity of collagen fibers, although there were conflicting reports regarding changes in fiber diameter (Duan et al., 2016; Ibrahim et al., 2019). Furthermore, the increased D-band periodicity may lead to improper collagen molecule accumulation, resulting in reduced mineralization of the dentine (Duan et al., 2016). Immunoelectron microscopy showed a higher expression of type III collagen in DGI-I dentine compared to normal dentine. Additionally, expression of type VI collagen was detected in DGI-I dentine, whereas it is not expressed in normal dentine (Waltimo et al., 1994). Studies have suggested that dysfunction in odontoblasts was associated with changes in dentine ultrastructure. On the one hand, gene mutations may directly result in odontoblast dysfunction (Liang et al., 2023). On the other hand, due to the intracellular accumulation of abnormal procollagen, odontoblasts may gradually expand and become dysfunctional. With the mineralization of secreted collagen fibers, the dilated odontoblasts would be enveloped, thereby impeding further collagen secretion (Majorana et al., 2010). In conclusion, in DGI-I, there are widespread alterations in the organization, structure, and orientation of collagen fibers and dentinal tubules, which impact dentine mineralization and reduce the mechanical strength of the teeth.

There is still controversy regarding whether the dentine-enamel junction (DEJ) is affected in DGI-I (Martín-Vacas et al., 2022). Limited research has been conducted on the impact on enamel, but it is generally believed that enamel remains unaffected (Devaraju et al., 2014). However, a study has surprisingly revealed that a heterozygous missense mutation in *COL1A2* can lead to glassy appearance of enamel, suggesting that type 1 collagen may play a role in enamel mineralization, resulting in enamel abnormalities in DGI-I (Kantaputra et al., 2018a). Similarly, Martin-Vacas (Martín-Vacas et al., 2022) also found structural changes in the enamel of DGI-I teeth, including fractures in enamel prisms and loss of prism patterns. It is important to note that it is still not clear whether the changes in enamel are caused directly by mutations of the *COL1* or indirectly by improper support of harmed dentine.

### 2.2 Dentinogenesis imperfecta type II (DGI-II)

DGI-II is a common inherited dental disorder with an estimated prevalence of 1/8000. Mutations in the *DSPP* have been identified as the pathogenic cause of DGI-II (Park et al., 2020). Dentine sialoprotein (DSP) and dentine phosphoprotein (DPP) are the main non-collagenous proteins in dentine, derived from enzymatic cleavage of DSPP. They function as regulating dentine mineralization initiation, collagen mineralization and maturation processes (Maciejewska and Chomik, 2012). The *DSPP* gene consists of 5 exons and 4 introns. Exon 2 encodes the signal peptide, exons 2-4 encode the N-terminal of DSP, and exon 5 encodes the C-terminal of both DSP and DPP protein (Ritchie, 2018). Over 50 *DSPP* gene mutation sites associated with DGI-II have been reported. *DSP* mutations commonly occur as missense and nonsense mutations, while the coding region for DPP is frequently affected by mutations in the signal peptide coding sequence and frameshift mutations.

Mutations in the signal peptides of *DSPP* often hinder protein translocation to the endoplasmic reticulum, thereby affecting the subsequent transport, modification, and secretion of DSP and DPP. Due to the reduced levels of DSP and DPP in the extracellular matrix, the deposition and mineralization of dentine were impaired (Maciejewska and Chomik, 2012). A study has documented a missense mutation (c.15C>T) in exon 2 of *DSPP* in a severe DGI-II Central American family, resulting in the substitution of leucine with alanine and potentially altering the structure of the signal peptide, leading to the occurrence of DGI (Malmgren et al., 2004).

The N-terminal of the DSP protein contains a highly conserved tripeptide domain composed of isoleucine, proline, and valine, which is crucial for maintaining the biological function of DSPP. When mutations occur in this region, the cleavage of the signal peptide and protein processing will be interrupted (von Marschall et al., 2012). The residues P 17 and V18 are speculated to be mutation hotspots that affect DSPP function. Here, we list the different mutation sites found in the *DSP* sequence that disrupt dentine formation (Table 2).

**TABLE 2:** Mutations in the coding sequence of the signal peptide, *DSP* and *DPP* associated with DGI-II.

Gene	Location	cDNA	Protein	Mutation class	Enamel affected	DEJ affected	References
Signal peptide	Exon 2	c.15C>T	p.(Ala15Val)	Missense	-	-	Malmgren et al. (2004)
*DSP*	Exon 2	c.49C>T	p.(Pro17Ser)	Missense	Thin and hypoplastic	Flat	Hart and Hart (2007), Zhang et al. (2007), Taleb et al. (2018), Qu et al. (2009)
*DSP*	Exon 2	c.49C>A	p.(Pro17Thr)	Missense	-	-	Xiao et al. (2001)
*DSP*	Exon 2	c.50C>T	p.(Pro17Leu)	Missense	-	-	Li et al. (2012), Porntaveetus et al. (2019), Lee et al. (2013), Hu et al. (2018)
*DSP*	Intron 2	c.52-1G>A	p.(Val18_Gln45del)	Splice site	-	-	Liu et al. (2016)
*DSP*	Intron 2	c.52-3C>A	p.(Val18_Gln45del)	Splice site	-	-	Holappa et al. (2006), Lee et al. (2011a), Wang et al. (2009)
*DSP*	Intron 2	c.52-3C>G	p.(Val18_Gln45del)	Splice site	-	-	Li et al. (2012), Lee et al. (2011a), Kim et al. (2004)
*DSP*	Intron 2	c.52-2A>G	p.(Val18_Gln45del)	Splice site	Thin and hypoplastic	Flat	Taleb et al. (2018)
*DSP*	Exon 3	c.52G>T	p.(V18F + p.V18_Q45del)	Missense	Enamel defects	Flat and wilder	Holappa et al. (2006), Lee et al. (2011a), Kim et al. (2004), Kim et al. (2005), Song et al. (2006)
*DSP*	Exon 3	c.53T>A	p.(Val18Asp)	Missense	Enamel defects	-	Kida et al. (2009), Lee et al. (2009), Lee et al. (2011b)
*DSP*	Exon 3	c.53T>G	p. (Val18Gly)	Missense	Enamel defects	-	Du et al. (2021)
*DSP*	Exon 3	c.133C>T	p.(Gln45X)	Nonsense	-	-	Song et al. (2006), Zhang et al. (2001)
*DSP*	Intron 3	c.135 + 2T>C	Splice+2T>C	Splice site	-	-	Zhang et al. (2011)
*DSP*	Exon 3	c.135G>T	p.(Gln45His)	Missense	Thin and hypoplastic	Flat	Taleb et al. (2018)
*DSP*	Intron 3	c.135 + 3A>G	p.(Val18_Gln45del)	Splice site	-	-	Bai et al. (2010)
*DSP*	Intron 3	c.135 + 1G>A	p.(Val18_Gln45del)	Splice site	-	-	Xiao et al. (2001)
*DSP*	Intron 3	c.135 + 1G>T	p.(Val18_Gln45del)	Splice site	-	-	McKnight et al. (2008a)
*DSP*	Exon 4	c.202A>T	p.(Arg68Trp)	Missense	-	-	Malmgren et al. (2004), Holappa et al. (2006)
*DPP*	Exon 5	c.1915_1918delAAGT	p.(Lys629GlnfsX674)	Frameshift	Uneven enamel surface	-	Porntaveetus et al. (2018)
*DPP*	Exon 5	c.2272delA	p.(Ser758AlafsX554)	Frameshift	-	-	McKnight et al. (2008a)
*DPP*	Exon 5	c.2349delT	p.(Ser783ArgfsX531)	Frameshift	-	-	Nieminen et al. (2011)
*DPP*	Exon 5	c.2525delG	p.(Ser842ThrfsX471)	Frameshift	-	-	McKnight et al. (2008a)
*DPP*	Exon 5	c.2593delA	p.(Ser865fsX1313)	Frameshift	-	-	Song et al. (2008)
*DPP*	Exon 5	c.2666delG	p.(Ser889ThrfsX425)	Frameshift	-	-	Nieminen et al. (2011)
*DPP*	Exon 5	c.2684delG	p.(Ser895MetfsX1313)	Frameshift	-	-	Song et al. (2008)
*DPP*	Exon 5	c.2684delG	p.(Ser895MetfsX418)	Frameshift	-	-	Li et al. (2017)
*DPP*	Exon 5	c.2688delT	p.(Asp896GlufsX418)	Frameshift	No obvious changes	-	Park et al. (2020), Lee et al. (2011c)
*DPP*	Exon 5	c.3438delC	p.(Asp1146fsX1313)	Frameshift	-	-	Song et al. (2008)
*DPP*	Exon 5	c.3504_3508dup	p.(Asp1170AlafsX146)	Frameshift	-	-	Yang et al. (2016a)
*DPP*	Exon 5	c.3509_3521del13bp	p.(Asp1170AlafsX139)	Frameshift	-	-	Li et al. (2017)
*DPP*	Exon 5	c.3546_3550delTAGCAinsG	p.(Asp1182fsX1312)	Frameshift	-	-	Song et al. (2008)
*DPP*	Exon 5	c.3560delG	p.(Ser1187MetfsX127)	Frameshift	-	-	Lee et al. (2011c)
*DPP*	Exon 5	c3582_3591delCAGCAGCGAT	p.(Asp1194GlufsX117)	Frameshift	-	-	Nieminen et al. (2011)
*DPP*	Exon 5	c.3625_3700del76bp	p.(Asp1209AlafsX80)	Frameshift	-	-	Nieminen et al. (2011)
*DPP*	Exon 5	c.3676delA	p.(Ser1226Alafs X88)	Frameshift	Enamel defects	-	Bloch-Zupan et al. (2016)

(“-” refers to not mentioned in the corresponding literature).

DPP is a highly hydrophilic acidic protein rich in Asp-Ser-Ser. The repetitive arrangement of carboxyl-phosphate structures at both ends provide cross-linking, which facilitates crystal nucleation and mineral formation (George et al., 1996). Gene site mutations encoding DPP result in frameshift mutations. Different mutation sites lead to different manifestations of DGI-II, suggesting a genotype-phenotype correlation. Changes in protein hydrophilicity are believed to be the cause of phenotypic diversity (Lee et al., 2019; Yang J. et al., 2016) (Table 2).

The typical histological features of DGI-II include the disappearance of the scalloped DEJ, irregular morphology of dentinal tubules and collagen fibers, decreased mineralization, occasional presence of interglobular dentine, and rarely accompanied by enamel changes (Taleb et al., 2018). In early studies, DEJ defects were believed to be the cause of enamel fragmentation (Wieczorek and Loster, 2013). However, subsequent research has shown that even if the DEJ was normal, the enamel in DGI-II patients was still prone to delamination. Additionally, the elastic modulus, hardness, and mineral density of DGI-II dentine were reduced, which was at least partially responsible for tooth fractures and attrition in DGI teeth (Park et al., 2020; Porntaveetus et al., 2018; Mao et al., 2021). Surprisingly, literature has reported the presence of primary hypoplastic enamel defects in DGI-II patients (Du et al., 2021). Similar enamel defects have also been found in two other DGI-II pedigrees of Korean and Caucasian descent (Lee SK. et al., 2011; Wang et al., 2011). It is speculated that *DSPP* is not only expressed in odontoblasts but also transiently expressed in ameloblasts. Although the dental pulp of DGI-II affected teeth may appear completely resorbed in X-ray, there may still exist interconnected vascular networks, providing a pathway for oral bacteria leading to periradicular abscesses (Davis et al., 2015).

Several studies have shown changes in the elemental composition of DGI-II dentine. Park et al. (Park et al., 2020) reported that compared to normal dentine, DGI-II dentine had lower levels of magnesium (Mg), while there was no difference in other elements such as sodium (Na), calcium (Ca) and phosphorus (P). X-ray microanalysis revealed that compared to normal primary molars, DGI-II primary molars showed decreased levels of Mg and carbon (C) and increased levels of oxygen (O) and Na. It was proposed that Na may be an important element in distinguishing DGI-II dentine from normal dentine (Sabel et al., 2020). Energy-dispersive spectroscopy analysis showed that Ca content and Ca/P ratio in DGI-II teeth were higher than in normal teeth (Du et al., 2021). The reasons for the varying results in elemental content in DGI-II are not yet clear. One possible explanation is that primary and permanent teeth have different elemental compositions, suggesting that the levels of calcium or phosphorus may be age-related.

### 2.3 Dentinogenesis imperfecta type Ⅲ (DGI-Ⅲ)

Due to the possibility that DGI-III and DGI-II may only differ in degree, there are few literature just focus on the pathogenesis and histopathological changes of DGI-III. Previous researches believed that *DSPP* mutations were the only cause of DGI-III (Table 3). It has been reported that a combined mutations including a 36-bp deletion together with an 18-bp insertion occurred at exon 5 could contribute to a shortening of the highly conserved COOH terminus, thereby altering the carboxyl-phosphate group structure of the DPP protein. As a result, the initiation, formation and maturation of dentine crystals are impaired (Dong et al., 2005). Mutations (c.52-2A>G, p. V18_Q45del; c.53T>A, p. V18D), which occurred in intron 2 and exon 3 respectively, were also reported to show a DGI-III-like pattern, including enlarged pulp chambers, root canals associated with attrition of primary teeth (Wang et al., 2009; Li et al., 2017). Interestingly, two families with the same mutation (c.52G>T, p. V18F) showed two different phenotypes of DGI-II and DGI-III, respectively. This may suggest that DGI-II and DGI-III were not separate diseases but phenotypic variation of the same disease (Kim et al., 2005). As a more severe type of DGI, DGI-III showed fewer dentinal tubules on the surface of fractured dentine. Furthermore, calcospherites at the calcification front were more irregular or even absent in the DGI-III compared to DGI-II (Levin et al., 1983).

**TABLE 3:** Mutations in the coding sequence of *DSPP* associated with DGI-Ⅲ.

Location	cDNA	Protein	Mutation class	Enamel affected	DEJ affected	References
Intron 2	c.52-2A>G	p.(Val18_Gln45del)	Splice site	-	-	Li et al. (2017)
Exon 2	c.52G>T	p.(Val18Phe)	Missense	-	-	Kim et al. (2005)
Exon 2	c.53T>A	p.(Val18Asp)	Missense	-	-	Lee et al. (2009)
Exon 3	c.133C>T	p.(Gln45X)	Nonsense	Enamel defects	Flat	Song et al. (2006)
Exon 5	c.3599_3634 del 36bp	Del1160-1171	Frameshift	-	-	Dong et al. (2005)
Exon 5	c.3715_3716 ins 18bp	Ins1198-1199	Frameshift	-	-	Dong et al. (2005)

(“-” refers to not mentioned in the corresponding literature).

### 2.4 Dentine dysplasia type Ⅰ (DD-Ⅰ)

DD-I is characterized by short roots, loose teeth and pain associated with numerous periapical radiolucencies in non-carious teeth. Carroll et al. classified DD-I into four subtypes based on radiographic features (O Carroll et al., 1991). DD-Ia is the most severe, characterized by complete absence of root canal and root formation, with no evidence of caries in the surrounding region. DD-Ib shows short roots with a “crescent-shaped” or “horseshoe-shaped” pulp chamber, with radiolucencies visible around the root apex. DD-Ic exhibits half the normal length of roots and distinct “new-moon-shaped” pulp chambers. In DD-Id, the root length is normal, and the pulp chamber and pulp calculi are visible (O Carroll et al., 1991; Chen et al., 2019a).

Studies have proposed several potential mechanisms to be responsible for DD-I. During tooth development, the epithelial root sheath prematurely invaginated into the dental papilla, resulting in abnormal root dentine formation. Additionally, abnormal interactions between ameloblasts and odontoblasts could lead to abnormal odontoblast function in DD-I patients (Alhilou et al., 2018). Recently, three mutant genes-*SMOC2*, *VPS4B*, and *SSUH2*-were found to be closely associated with DD-I in three different families (Chen et al., 2019a) (Table 4).

**TABLE 4:** Pathogenic gene mutations associated with DD‐I.

Gene	Location	cDNA	Protein	Mutation class	Morphology of root	References
*SMOC2*	Intron 1	c.84 + 1G>T	Splice+1G>T	Splice site	Extremely short	Bloch-Zupan et al. (2011)
*SMOC2*	Exon 1	c.24G>A	p.Trp8Ter	nonsense	Short and blunt	Ruan and Duan (2022)
*VPS4B*	Intron 7	IVS7+46C>G	Splice+46C>G	Splice site	Short, blunt and malformed	Yang et al. (2016b)
*SSUH2*	Exon 2	c.353C>A	p.(Pro118Gln)	Missense	Short, blunt and malformed	Xiong et al. (2017)

SMOC2 belongs to the BM-40 family and is involved in regulating cell-matrix interactions (Vannahme et al., 2003), particularly in bone mineralization. A splicing mutation (c.84 + 1G>T) of *SMOC2* was detected in a family, in which the homozygous exhibited DD-I-like phenotype with short roots, while the heterozygous showed normal teeth. Knockdown of the *smoc2* gene in zebrafish leads to abnormal tooth development by regulating the expression of *dlx2*, *bmp2*, and *pitx2* (Bloch-Zupan et al., 2011).

Vacuolar protein sorting 4B (VPS4B) is a versatile protein widely expressed in pulp tissue. *In vivo* and *in vitro* studies demonstrated that VPS4B regulated odontoblast differentiation and root formation through the Wnt/β-catenin signaling pathway (Yang Q. et al., 2016; Li et al., 2020). It is speculated that mutations of *VPS4B* may affect the spatial distribution of DSPP and COL1, inhibiting normal mineralization of dentine and cementum, and ultimately leading to abnormal root development (Chen et al., 2019b).

SSUH2 is a nuclear protein with an unclear function (Xiong et al., 2017). Heterozygous missense mutation (c.353C>A, p. P118Q) of the *SSUH2* may result in protein dysfunction, leading to abnormal dentine hyperplasia and a narrowed dental pulp chamber (Xiong et al., 2017). Mechanically, SSUH2 might form a transcriptional complex by binding upstream regulators associated with the bone morphogenetic protein (BMP) or DSPP signaling pathway to act as a chaperone protein in the nucleus, regulating the differentiation of ameloblast and odontoblast (Xiong et al., 2017).

There is relatively limited literature on the pathogenesis of DD-I, and the relationship between the three mentioned genes and DD-I is only based on case studies. The mechanisms of these related genes in dentine mineralization and tooth root formation still need further exploration.

Histologically, the morphology and color of the DD-I tooth crown appeared normal. However, the roots were often short or absent with calcified dentine filling in the root canal. The dentinal tubules were sparse and narrow, containing spherical calcites. Periapical lesions showed cyst-like alteration. In addition, changes including “teardrop-shaped” lacunae near the cervical enamel, rodless enamel, flat DEJ, thinner dentine and disordered collagen fibers could be found in DD-I teeth (Devaraju et al., 2014; Alhilou et al., 2018).

### 2.5 Dentine dysplasia type Ⅱ (DD-Ⅱ)

*DSPP* has been recognized as the pathogenic gene of DD-II. Specifically, exon 2 encoding signal peptides and exon 5 encoding DPP protein were considered the main mutation sites. Furthermore, there was a genotype-phenotype correlation in codons encoding DPP. For example, the frameshift mutations at the N-terminal were related to DD-II, while mutations at C-terminal were more likely to lead to DGI-II (Lee et al., 2019) (Table 5).

**TABLE 5:** Mutations in the coding sequence of *DSPP* associated with DD-Ⅱ.

Location	cDNA	Protein	Mutation class	References
Exon 2	c.16T>G	p.(Tyr6Asp)	Missense	Rajpar et al. (2002)
Intron 2	c.52-6T>G(IVS2-6T>G)	p.(Val18_Gln45del)	Splice site	Lee et al. (2008)
Exon 5	c.1686delT	p.(Asp562GlufsX752)	Frameshift	Nieminen et al. (2011)
Exon 5	c.1830delC	p.(Ser610ArgfsX704)	Frameshift	Nieminen et al. (2011)
Exon 5	c.1870_1873delTCAG	p.(Ser624TyrfsX687)	Frameshift	McKnight et al. (2008a)
Exon 5	c.1874_1877delACAG	p.(Asp625AlafsX687)	Frameshift	Li et al. (2017)
Exon 5	c.1918_1921delTCAG	p.(Ser640TyrfsX671)	Frameshift	McKnight et al. (2008a)
Exon 5	c.1918-1921delTCAG	p.(Ser640TyrfsX673)	Frameshift	Nieminen et al. (2011)
Exon 5	c.1922-1925delACAG	p.(Asp641AlafsX672)	Frameshift	Nieminen et al. (2011)
Exon 5	c.2040delC	p.(Ser680fsX1313)	Frameshift	Song et al. (2008)
Exon 5	c.2063delA	p(Asp688ValfsX626)	Frameshift	Nieminen et al. (2011)
Exon 5	c.2134delA	p.(Ser712AlafsX602)	Frameshift	Lee et al. (2019)
Exon 5	c.3135delC	p.(Ser1045ArgfsX269)	Frameshift	Yang et al. (2016a)
Exon 5	c.3141delC	p.(Ser1047fsX223)	Frameshift	McKnight et al. (2008b)
Exon 5	c.3179delG	p.(Ser1060ThrfsX254)	Frameshift	Lee et al. (2019)
Exon 5	c.3480_3481insCTGCT	p.(Asp1161LeufsX155)	Frameshift	Lee et al. (2019)

In DD-II patients, the tooth crown and root showed discrete histological features. Specifically, the crown dentine appeared normal, while a superficial layer of dense, amorphous tubular dentine in the roots. Numerous pulp stones composed of amorphous calcified masses can be observed in the pulp tissue. As a consequence, the pulp chamber would gradually obliterate with age (Perlea et al., 2018). The literature about DD-II is relatively few since DD-II is a mild form of DD, whose clinical symptoms are mild, making it difficult to attract the attention of the patients and dentists.

## 3 Management of hereditary dentine disorders

Hereditary dentine disorders are genetic developmental abnormalities of dental hard tissues that can lead to tooth discoloration, excessive wear, looseness, and tooth loss. Reduced chewing function and decreased occlusal height might perform adverse effects on the development of the craniofacial skeleton and muscles, and may even result in temporomandibular disorder (TMD), potentially leading to malocclusion (Crothers, 1992). Besides, as mentioned earlier, DGI-I, as a typical oral manifestation of OI, could provide clues for orthopedics, pediatrics, and other disciplines to detect and intervene in systemic diseases early. Therefore, early diagnosis, optimal treatment, and follow-up are recommended to prevent further damage.

There is growing consensus that interdisciplinary teamwork is essential for achieving optimal treatment. It is recommended that the team includes at least one pediatric dentist, periodontist, orthodontist, prosthodontist, oral surgeon, clinician and genetic counselor. Treatment should be initiated early and carried out in stages if necessary (Rousseau et al., 2018). The purpose of early intervention and treatment goals is to prevent dental caries and dentine wear, avoid premature tooth loss, restore the chewing function and aesthetic, and maintain vertical occlusal distance (Delgado et al., 2008), as well as identify and/or exclude associated systemic syndromes. Currently, there are no specific guidelines on when to initiate treatment, but considering the adverse impacts on the teeth, early intervention is necessary to prevent the escalation of complexity and risk. A case report has shown that dental crown treatment was performed under general anesthesia on a 20-month-old patient, effectively preventing severe tooth wear and achieving favorable long-term outcomes (Sapir and Shapira, 2001).

### 3.1 Tooth wear management

One of the main concerns for patients with DGI is tooth wear. Due to developmental defects and low mineralization of dental hard tissues, the enamel is easily peeled off, exposing fragile dentine and causing varying degrees of tooth wear. Accordingly, dental restorative treatment is essential to protect the remaining dental tissues, restore chewing function and maintain bite height. According to the tooth wear index classifications proposed by Smith et al. (Smith and Knight, 1984), Grade I and Grade II (enamel and dentine wear with an exposed area less than 2 mm^2) can be restored using composite resin bonding to regain tooth aesthetics and function (Yuan and Chen, 2023; Loomans and Opdam, 2018). For Grade III and Grade IV wear in primary teeth (exposed dentine area >2 mm^2 or pulp exposure), it is recommended to use stainless steel preformed crowns to prevent further wear of the tooth crown (Crawford et al., 2007). If severe wear occurs in primary teeth, overlay dentures can be considered for restoration (Yuan and Chen, 2023; Chen et al., 2019a). In the mixed-dentition stage, close monitoring of the eruption of permanent teeth is necessary. Stainless steel crowns and composite materials, such as strip crowns, indirect resin crowns, or polycarbonate crowns can be used to cover permanent molars and incisors, respectively (Shi et al., 2020). For permanent teeth with Grade III and Grade IV wear requiring occlusal reconstruction to restore vertical height, options such as full crowns or onlays can be considered. In cases of severe tooth wear, the use of overlay or implant-supported prostheses for restoration may be considered (Syriac et al., 2017).

### 3.2 Tooth discoloration management

Teeth affected by DGI exhibit discoloration ranging from pale yellow, brown to bluish brown, especially in the anterior area, which greatly impacts patients’ smile aesthetics and mental health. Therefore, the restorations of anterior tooth should consider both aesthetics and function. For mild wear and discoloration of teeth, veneer restorations can achieve satisfactory results. However, it should be noted that there is a risk of enamel delamination due to decreased mechanical bonding strength between enamel and dentine. When necessary, full crown restorations can be considered (Shi et al., 2020). Digital technology is beneficial for achieving aesthetic restorations in the anterior region. It allows precise design of the restoration’s size, shape, and coordination with surrounding gingival margin. For patients with clinically short crowns or poor gingival margin contours, crown lengthening surgery is also crucial for achieving aesthetic outcomes (Kalsi et al., 2020).

### 3.3 Dental pulp and periapical management

When severe tooth wear leads to pulp exposure and inflammation of the pulp and periapical tissues, root canal treatment becomes necessary to alleviate pain, eliminate inflammation and prolong teeth lifespan. However, before carrying out dental treatment, detailed and comprehensive clinical and radiological examinations must be conducted to evaluate the morphology of the pulp cavity, length of root and status of periapical tissues. The narrow or even absent pulp chamber and root canal, and the presence of pulp stones undoubtedly increase the difficulty of clinical procedures. In such cases, the use of cone-beam computed tomography (CBCT) and microscopy techniques can help improve the success rate (Buchanan et al., 2021). Additionally, when the root canal length is sufficient, apical surgery and retrograde filling have been reported as treatment options (Ravanshad and Khayat, 2006).

### 3.4 Dental restoration and implant management

When encountered with severe dental inflammation, heavy tooth defects and excessive tooth mobility (due to absent or extremely short roots appearing in certain subtypes of DGI and DD), leading to premature tooth loss or poor dental prognosis, extraction followed by restorative or implant treatment is the best solution. During the growth and development period, the best choice is dentures to stimulate and maintain the alveolar bone mass at the site of missing teeth (Barron et al., 2008). As the jawbone develops, it is recommended to regularly consult with the dentist to adjust the denture, making it adapting to the dental arch at any time. After the growth and development are completed, restorative treatment and dental implantation can be chosen. A comprehensive clinical and radiological examination is necessary to evaluate the bone quality and quantity in the area of missing teeth. When the periodontal tissues are in good condition and interdental distance is sufficient, dental implantation could be a popular therapy. It should be noted that the implantation treatment of DGI-I patients with moderate to severe OI should be cautious, as they may face a higher failure rate due to poor bone remodeling ability (Rousseau et al., 2018).

### 3.5 Malocclusion management

Studies have found that individuals with DGI-I are more prone to Class III malocclusion, suggesting the need for early orthodontic intervention (Kunkel et al., 2019). Although maxillary bone development is compromised in individuals with DGI, especially those with type III and type IV OI, traditional methods such as anterior traction and rapid maxillary expansion may not be effective due to limited growth potential of sutures and high bone fragility. In cases of severe skeletal malocclusion, orthognathic surgery and distraction osteogenesis may be considered when necessary (Kunkel et al., 2019).

In addition, orthodontic treatment for DD-I patients is challenging, as short roots make teeth difficult to move under orthodontic forces, and inappropriate orthodontic forces may lead to further absorption of the tooth roots. Therefore, comprehensive examination and sufficient doctor-patient communication are necessary before starting orthodontic treatment. Attention should be paid to controlling orthodontic force to prevent adverse consequences from occurring (Papagiannis et al., 2021).

### 3.6 General management and genetic counseling

Notablely, hereditary dentine disorders could be an independent phenotype or a part of systemic disorders. In addition to proverbial OI, it has been reported that systemic diseases including Schimke immuno-osseous dysplasia, odontochondrodysplasia, hypophosphatemic rickets and Ehlers-Danlos syndrome are also accompanied by clinical phenotypes of noticeable dentine defects (Yuan and Chen, 2023; Su et al., 2023). Abnormal dentine phenotype may reflect the health status of teeth, skeletal system, and even the entire body. Therefore, clinical physicians and genetic counselors must be alert to the emergence of potential complex diseases or systemic disorders. For abnormalities in the skeletal system, clinician need to adopt appropriate bone management strategies as early as possible, such as professional physical therapy, surgical corrective surgery, rehabilitation training, *etc.*, to maximize the preservation of patients’ independent living abilities and improve their life qualities (Jovanovic et al., 2022). Genetic counseling is also an important part, using molecular genetic diagnostic methods to make the best decisions based on the identified pathogenic genes behind these diseases.

## 4 Conclusion

So far, the etiology, clinical features, and histopathological characteristics of hereditary dentine diseases have not been fully elucidated. Techniques such as atomic force microscopy and Wallace indentation provides convenience for exploring the mechanical properties and histological changes of affected teeth. As more and more cases are discovered, the genetic spectrum is gradually being delineated. Further research on the relationship between genotypes and phenotypes provides clues for genetic counseling. Due to the heterogeneity of tooth damage types and severity, treatment methods also exhibits personalized and diverse characteristics. Early detection, diagnosis, and multi-disciplinary treatment can help develop optimal therapy plans for patients, maximizing facial aesthetics and oral function, while reducing social and psychological burdens.
